# Suicide preceded by health services contact – A whole-of-population study in New Zealand 2013-2015

**DOI:** 10.1371/journal.pone.0261163

**Published:** 2021-12-20

**Authors:** Annie Chiang, Janine Paynter, Richard Edlin, Daniel J. Exeter

**Affiliations:** 1 Epidemiology and Biostatistics, School of Population Health, The University of Auckland, Auckland, New Zealand; 2 General Practice and Primary Healthcare, School of Population Health, The University of Auckland, Auckland, New Zealand; 3 Health Systems, School of Population Health, The University of Auckland, Auckland, New Zealand; University of the Witwatersrand, SOUTH AFRICA

## Abstract

New Zealand’s rate of suicide persistently exceeds the global average. The burden of suicide in New Zealand is disproportionately borne by youth, males and Māori (NZ indigenous people). While the demographic characteristics of suicide decedents are established, there is a need to identify potential points of contact with health services where preventative action could take place. This paper aims to determine if suicide deaths in New Zealand were likely to be preceded by contact with health services, and the type and time frame in which these contacts took place. This study utilised a whole-of-population-cohort of all individuals age 15 years and over, who were alive on March 5^th^ 2013, followed up to December 2015. Associations between the odds of suicide, demographic factors, area-based deprivation, and the timing of last contact with primary, secondary, and tertiary services were analysed using univariate and multivariate logistic regression. Contact with a health service in the 6 Months prior to death was associated with the highest odds of suicide. Over half of the suicide decedent population (59.4%) had contacted primary health services during this period. Large proportions of the suicide decedent population contacted secondary and tertiary services in the 6 Months prior to death, 46.5% and 30.4% respectively. Contact with primary, secondary and tertiary services in the prior 6 Months, were associated with an increased odds of suicide of 2.51 times [95% CI 2.19–2.88], 4.45 times [95% CI 3.69–4.66] and 6.57 times [95% CI 5.84–7.38], respectively, compared to those who had no health services contact.

## Introduction

In 2018, the Government Inquiry into Mental Health and Addictions described the rate of suicides in New Zealand as “persistently high” [1]. The annual provisional suicide statistics released by the Coronial Services of New Zealand showed a steady increase in national suicide rates of 11.37 suicides per 100,000 in 2013 to, in their most recently-available data, 13.93 suicides per 100,000 in 2019 [2]. Both these figures exceed the WHO’s 2016 estimate [3] for the global suicide rate of 10.6 per 100,000.

Suicides in New Zealand (NZ) are disproportionately high amongst youth, males, and Māori (NZ indigenous people). Those aged 15–24 years old, account for approximately 25% of suicide deaths each year [4], resulting in New Zealand having one of the highest rates of youth suicide rates in the Organisation of Economic Cooperation and Development (OECD) [5]. In 2013, the youth suicide rate in New Zealand for female youth was 11.7 per 100,000 and 24.1 per 100,000 for male youth, representing the highest and third highest rates in the OECD respectively [6]. Males in NZ are 2.5 times more likely to die of suicide compared to females, despite females being twice as likely to be hospitalised for self-harm or attempted suicide [6]. There are also ethnic disparities in the rates of suicide in New Zealand. For example, the Māori suicide rate for 2012 to 2016 was 17.1 per 100,000, while rates for Pacific, Asian and Other ethnicities was 8.1, 4.2 and 11.3 per 100,000, respectively [7].

New Zealand’s health system is predominantly publicly funded, with a mix of public, private and non-government service providers [8]. Primary care in New Zealand refers to community-based general practice services. These are typically private practices funded through capitation and fee-for-service. Primary care practices provide access to General Practitioners (GPs), nurses, pharmaceutical, diagnostic, therapeutic and support services. GPs are the most visited health professional in New Zealand, and act as gatekeepers to other specialist’s services. Secondary services in New Zealand refers to specialist services that are beyond the scope of general practice, and usually accessed through GP referral. Tertiary care generally refers to care requiring hospitalisation, such as for the treatment of serious or complex conditions.

The prevailing narrative that suicides are strongly associated with or precipitated by mental illness, although valid and important, has limited interest in research seeking to understand the risk of suicide among individuals who may not be accessing specialised mental health services. The narrative that suicide entails mental health disorders is problematic, since implementation suicide prevention strategies in Australia found that services faced challenges in providing care to those in crisis who were not diagnosed with a mental health disorder [9]. Prevention strategies should be an all-of-service approach, that meet a diverse needs of the population, rather than having intervention efforts directed exclusively towards those perceived to be in crisis [9, 10]. Existing literature on suicide risk, deaths resulting from suicide and health services contact have studied sub-populations, such as youth [11] or mental health services users [12, 13] or a single service type exclusively [14–16]. Instead of a mental health service or high-risk population approach, our study extends upon the existing literature relating to suicide and health services contact in New Zealand by using individual-level data within a whole-of-population study design. We investigate the contact of suicide decedents from 2013–2015 with publicly funded health services in three different settings. Our analyses adjust for demographic and socioeconomic characteristics that are well established as risk factors for suicide [17–24].

## Methods

### Data

Much of the data used in this study was collected through the New Zealand 2013 Census, which aimed to collect data from all residents in New Zealand on March 5, 2013, using self- or proxy-completed forms for each person and another for each dwelling. This study utilised the Statistics New Zealand Integrated Data Infrastructure (IDI), which contains anonymised microdata on individuals and households collected by government and non-governmental agencies. Of relevance to this study, the IDI includes both the National Census 2013 dataset and Ministry of Health datasets (Primary Health Organisation, National Non-Admitted Patient Collection and Publicly Funded Hospital Discharges). The Ministry of Health also collects data from private hospital discharges, though we have excluded this from our analysis. The private system in New Zealand is supplementary to the universal coverage of the public system by providing non-urgent, elective treatments. With the relatively low uptake of private insurance by New Zealand, that addition of private hospital discharges is highly unlikely to affect the outcome of this study, and was therefore excluded [25].

The IDI data repository is administered by Statistics New Zealand, which ensures the confidentiality of data stored within the databases and any resulting publications [26]. To gain access to the IDI datasets, all researchers and projects must be vetted to ensure that the research is in the public interest. All data stored within the IDI have identifying details such as dates of birth, identification numbers and address removed before they are made accessible to researchers in Statistics New Zealand’s secure data labs. In compliance with the Statistics New Zealand’s requirements (the Five Safes Framework), all outputs seen in the results have been checked to ensure that that individuals cannot be identified and were approved for release by Statistics New Zealand [26].

### Study design

This observational study used a whole-of-population cohort, where cases are defined as all those who died of suicide between March 2013 and December 2015, while controls were those who were alive at the end of this study period. Causes of death were recorded using the International Statistical Classification of Diseases and Related Health Problems, Tenth Revision, Australian Modification (ICD-10-AM) [27]. Suicides were deaths coded as either X60–X80 (external causes of morbidity and mortality resulting from intentional self-harm), or as Y10–Y34 and Y87 where the injury cause of death was self-inflicted but there was insufficient evidence to determine if the event was accident, non-suicidal self-harm or suicide [19, 20, 24, 28].

In New Zealand, suicide statistics are provisionally reported by the Coronial Service, while the Ministry of Health reports official suicide statistics. The official statistics only include deaths where the suicidal intent of the decedent has been confirmed following a coronial investigation [29]. This process results in a delay in the release of the official statistics. At the time this study was conducted, the most up to date release of official suicide statistics were for the 2015 calendar year.

### Cohort construction

Our study cohort was defined as individuals aged 15 years and over who were alive on March 5^th^, 2013 (Census day) who were either alive at the end December 2015 or died as a result of suicide during this period. This age range includes those in the high risk age range of 15–24 years, whilst excluding younger people in whom determination of suicidal intent can be contentious [30]. Our study cohort was derived from the Census 2013 Individuals Dataset and supplemented with mortality datasets to capture all those that would have been alive on Census day. The study cohort included a total of 3,408,765 individuals.

### Demographic variables

**Fig 1:**
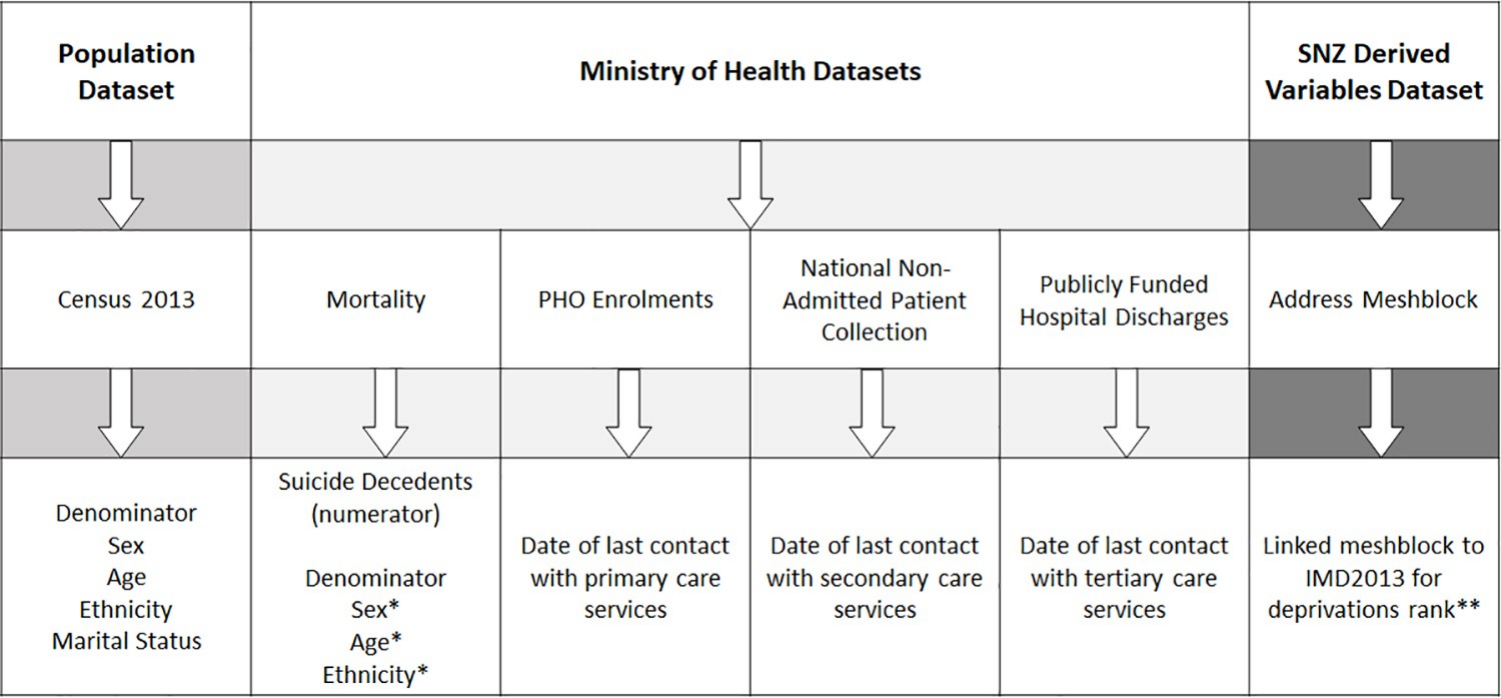
Flow diagram of the source dataset in the IDI (Top row), the specific data tables utilised (Middle Row) and the variables that were extracted for our analyses (Bottom Row). *Where data was not available in the Census dataset, this was supplemented from data in the mortality dataset. **Where address data was not available from either Census or mortality datasets, this was imputed from the SNZ derived variables dataset.

Demographic variables included sex, age and ethnicity and were derived from both Census and mortality datasets, as seen in Fig 1. Responses to Census 2013 were used to code sex as male or female. For individuals who were not enumerated in the Census, sex data was extracted from the mortality dataset. Age was coded into five-year bands, up to 75–79 years, with those aged 80 years and above combined. Prioritised Ethnicity was coded according to the Health Information Standards Organisation’s (HISO) Ethnicity Protocols [31], which allow up to six ethnic groups to be selected for each individual. The Prioritised Output reporting assigns individuals into the highest priority ethnicity group with which they identify; the priority order is Māori, Pacific, Asian, MELAA, Other, and then European [31]. The intent of the Prioritised Output protocol is to ensure that minority ethnic groups which are of policy importance, which is the case for Māori and suicide, are not swamped by the European majority. Marital status was categorised as single, married, civil union, separated, divorced, or widowed as per the question format in Census 2013. An additional category for “Undefined” marital status includes individuals who did not respond to the Census question, or the response was unidentifiable, or were a suicide decedent that was not enumerated in the Census. For unenumerated suicide decedents, no marital status data was available.

### Indices of multiple deprivation

We used the 2013 edition of the New Zealand Indices of Multiple Deprivation (IMD2013), an area-based measure of deprivation that comprises seven domains of deprivation: employment, income, crime, education, housing, health and access [32]. An advantage of the IMD2013 is that it provides an overall measure of deprivation while also enabling the testing of each domain of deprivation independently. The indicators used to construct each domain have also been tested to for their robustness in representing a particular form of deprivation [32].

To assign an area deprivation score to every participant, their address mesh block on Census day was matched to its corresponding data zone. Where mesh block data was missing from the Census dataset, this was imputed from the mortality dataset and subsequently from the Statistics New Zealand derived variable dataset if further imputation was required. Data zones are a geographic scale where each area contains an average of 712 people. This scale is large enough to represent neighbourhoods, whilst small enough to preserve homogeneity of the socioeconomic conditions experienced by residents [32]. A further strength of the IMD is its ability to exclude domains when outcomes of interest are also included in a domain. We therefore used the IMD2013 without the Health domain as the measure of deprivation as the Health domain comprises mortality data, as well as occurrence of select infectious and respiratory illnesses, cancers, and emergency admissions, whilst our study is interested in a broader range of health interactions. For the purposes of our analysis, we have coded the IMD2013 ranks into deciles, with Decile 1 being the least deprived, and Decile 10 the most deprived.

### Contact with health services

This study investigated all contacts in primary, secondary, and tertiary settings. Primary setting contact is captured through the Primary Health Organisations (PHO) dataset which records only the most recent consultation date in a primary setting, with this data being updated quarterly. These consultations could be provided by GPs or practice nurses, though given our aim is to determine the risk of suicide associated with the setting of contact, the distinction of health professional providing the service was not included in our study.

Two datasets were selected as proxies for secondary and tertiary care contact. These contacts include all reasons for contact, so will include but not be limited to those for mental health needs. Secondary contacts were defined as those that appeared in the National Non-Admitted Patient Collection (NNPAC) dataset that records medical and surgical outpatient care appointments, including non-scheduled visits to hospital emergency wards of less than three hours. We excluded events recorded as ’Did not attend’. Tertiary contacts were identified from the Publicly Funded Hospitalisations data set, which records all hospitalisations including both emergency (where duration of visit was greater than three hours) and elective care. We created binary variables for service contact and did not place any criteria on the nature of contact. Whilst adjusting for the reason for contact or the kind of care received would have enriched this study further, this data was not consistently available across all datasets. We calculated how many months elapsed between each type of health service contact and either the suicide event (for suicide decedents) or December 2015 (for those alive at the end of the study period). The time since last contact was categorised into groupings covering 0–6, 7–12, 13–18 and 19–24 Months. Due to confidentiality protocols that prevented the output of data with small cell counts, we were unable to report analyses for time periods shorter than 6 months. These periods were mutually exclusive, so that someone who had contacts at 5 and 9 Months would be coded as a “1” for contact within 0–6 Months and a “0” within 7–12 Months. In contrast, a person who only had a contact at 9 Months only would be coded as a as a “0” for contact within 0–6 Months and a “1” within 7–12 Months.

### Analysis

Our descriptive analyses involved univariate analyses of the association between demographic variables (age, sex, ethnicity, marital status, deprivation), time of last contact, and suicide status. We conducted univariate logistic regressions and used a Base Model comprising age, sex, ethnicity, marital status and IMD2013, then tested the effects of contact with each health service type independently. Statistical analyses were conducted using STATA MP 16. As such, the ’base’ model provides findings without the impact of contact being included and the three ’full’ models provide findings including contacts in primary, secondary, and tertiary care, respectively.

In our models, the reference group for sex was “female” as this group has a lower risk for suicide. The youngest age group, 15–19 years, was the reference age group, to show the odds of suicide change with increasing age. “European” was selected as the reference ethnic group as they are the majority ethnic group in NZ [33]. “Single individuals” was the reference group for marital status, as all other groups implied individuals were either presently or previously coupled. Odds of suicide associated with deprivation were compared to the least deprived group, IMD2013 Decile 1, as the risk of suicide is expected to be lowest in the least deprived group [7, 34]. For regressions that adjusted for the period of last contact, those without contact with the service type of interest was selected as the reference group. This allowed us to determine whether contact was associated with increased odds of suicide.

## Results

Of the 3,408,765 individuals in our dataset, 1,560 died because of suicide by the end of the study period. Of the suicide decedents 30.8% (480/1560) were absent from the Census 2013 dataset and appeared only within in the mortality dataset.

**Fig 2:**
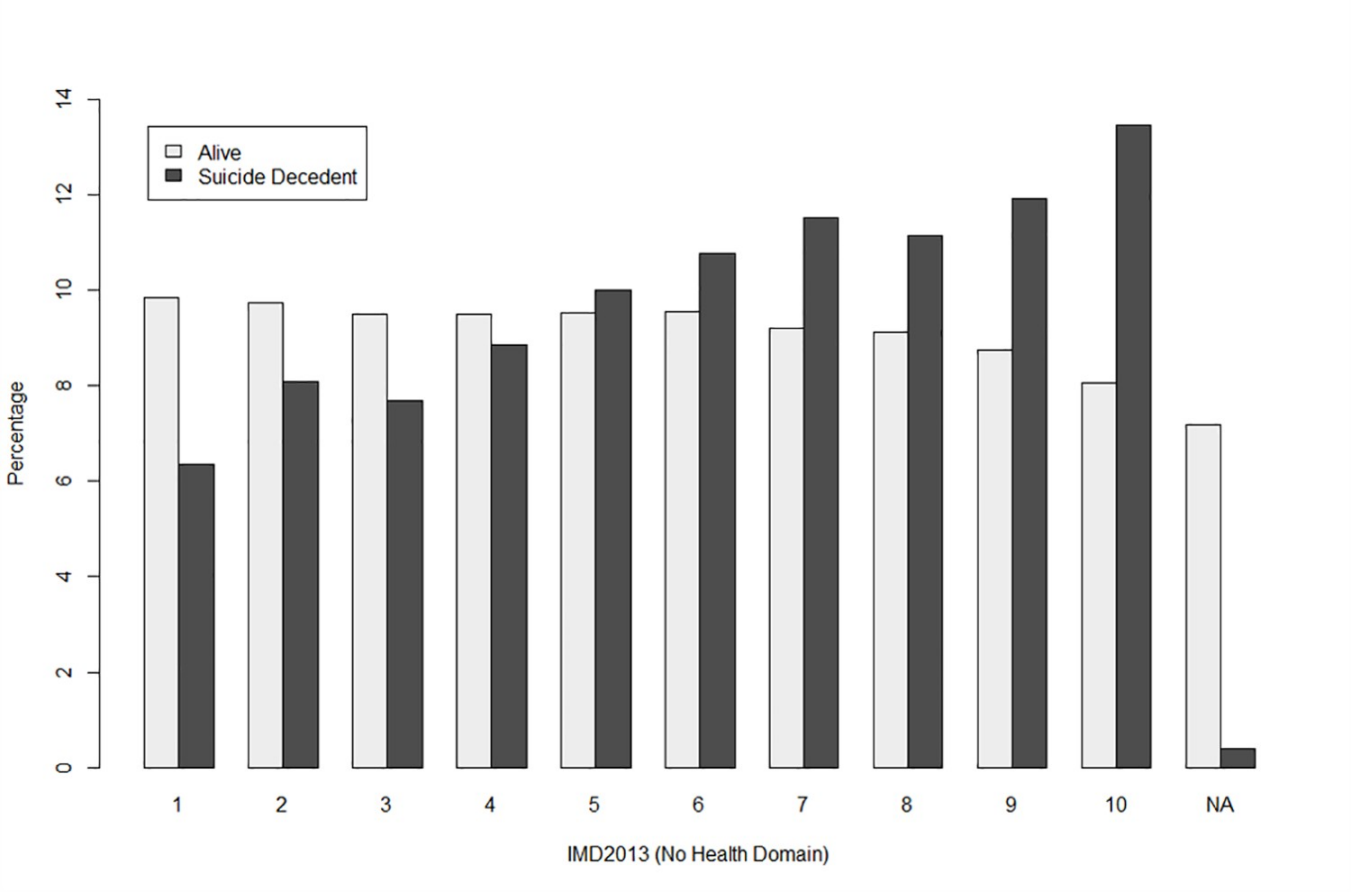
Distribution study cohort by status at the end of the study period and deprivation measured by IMD2013. IMD2013 Decile 1 represents the 10% least deprived areas in New Zealand and Decile 10 represents the most deprived.

Our results found that males were overrepresented in the suicide decedent population. Males accounted for 73.7% of suicide decedents but represented 48.1% of individuals alive at the end of the study period. Ethnic disparities are evident with overrepresentation of Māori and Europeans in the suicide decedent population. Māori account for 23.1% of suicide decedents but accounted for 12.3% of the population alive at the end of the study period. Pacific, Asian MELAA and Other ethnic groups comprise smaller proportions of the suicide decedent populations than their counterparts in the surviving population. In terms of marital status, those with an Undefined marital status (Not stated or did not fill in the census) were over-represented in the suicide decedent population, accounting for 34.8% of suicide deaths. Fig 2, demonstrates that the proportion of suicides increased with increasing levels of area-level deprivation.

Table 1 shows the period in which the last contact with health services occurred for our study population. In the 0–6 Months prior to suicide, 59.4% of suicide decedents had contact with primary health services, 46.5% contacted secondary health services, and 30.4% had contact with tertiary services. Comparatively, the proportion of those alive at the end of the study period who had health services contact were 39.6%, 21.2%, and 7.7% for primary, secondary, and tertiary services, respectively. Except for contact with in the 7–12 Month period prior to suicide, the proportion of suicide decedents that had contact with health services in the 7–24 Months prior to suicide broadly mirrored that of the population.

**Table 1:** Timing of last contact for suicide decedents and individuals alive at the end of the study period.

Service Type	Time period of last contact	Alive		Suicide Decedents	
		n	%	n	%
**Primary**	0–6 Months	1,349,925	39.6%	927	59.4%
	7–12 Months	846,015	24.8%	189	12.1%
	13–18 Months	250,269	7.3%	81	5.2%
	19–24 Months	127,485	3.7%	48	3.1%
	No contact	833,508	24.5%	315	20.2%
**Secondary**	0–6 Months	722,916	21.2%	726	46.5%
	7–12 Months	287,640	8.4%	135	8.7%
	13–18 Months	214,632	6.3%	90	5.8%
	19–24 Months	160,947	4.7%	63	4.0%
	No contact	2,021,067	59.3%	549	35.2%
**Tertiary**	0–6 Months	263,355	7.7%	474	30.4%
	7–12 Months	170,175	5.0%	105	6.7%
	13–18 Months	149,913	4.4%	81	5.2%
	19–24 Months	123,450	3.6%	60	3.8%
	No contact	2,700,315	79.3%	843	54.0%

Overall, Table 1 suggests that suicide decedents are more likely to have some of contact with health services in the two years prior to suicide compared to the general population who were alive at the end of the study period. In terms of primary services, only 20.2% of suicide decedents had no contact in the 24 Months prior to death, compared 24.5% of the general population who had no contact during the study period. Secondary and tertiary service contacts are less common among the general population, with 59.4% and 79.3% of the general population having had no contact with these service types, respectively, compared to just 35.3% and 54.0% suicide decedents.

Logistic regression was used to model the likelihood that suicide decedents were in contact with health services within 24 Months of their death, controlling for demographic factors. The unadjusted (univariate) analyses (Table 2) showed that males were approximately three times more likely to die of suicide compared to females. Decedents who were Māori, were separated divorced or in a civil union, had increased odds of dying from suicide compared to New Zealand Europeans, and those not in a relationship (i.e. Single) respectively. In addition, likelihood of suicide increased with increasing decile of socioeconomic deprivation.

**Table 2:** Univariate and adjusted odds ratios for base model and full model.

		Univariate	Base Model	Full Model
		(Unadjusted OR)	(Adjusted OR)	(Base Model +Any contact)
**Sex**	Female	*Reference*	*Reference*	*Reference*
	Male	3.02 (2.70–3.38)*	2.97 (2.65–3.32)*	3.41 (3.04–3.82)*
**Age Band**	15–19	*Reference*	*Reference*	*Reference*
	20–24	0.99 (0.80–1.22)	1.21 (0.97–1.50)	1.29 (1.04–1.60)*
	25–29	0.77 (0.61–0.97)*	1.08 (0.85–1.37)	1.14 (0.90–1.45)
	30–34	0.95 (0.76–1.19)	1.63 (1.30–2.04)*	1.62 (1.29–2.03)*
	35–39	0.85 (0.67–1.07)	1.55 (1.23–1.96)*	1.47 (1.16–1.85)*
	40–44	0.92 (0.74–1.15)	1.71 (1.37–2.13)*	1.53 (1.23–1.92)*
	45–49	0.95 (0.77–1.18)	1.77 (1.42–2.22) *	1.52 (1.22–1.90)*
	50–54	0.86 (0.69–1.08)	1.61 (1.28–2.04)*	1.31 (1.03–1.65)*
	55–59	0.85 (0.67–1.07)	1.63 (1.28–2.08)*	1.24 (0.97–1.59)
	60–64	0.59 (0.45–0.78)*	1.17 (0.89–1.55)	0.84 (0.63–1.11)
	65–69	0.39 (0.28–0.54)*	0.77 (0.54–1.09)	0.53 (0.37–0.75)*
	70–74	0.50 (0.35–0.70)*	0.97 (0.68–1.39)	0.63 (0.44–0.90)*
	75–79	0.58 (0.40–0.85)*	1.08 (0.73–1.60)	0.67 (0.45–0.99)*
	80+	0.92 (0.68–1.23)	1.52 (1.11–2.10)*	0.94 (0.68–1.30)
**Ethnicity**	European	*Reference*	*Reference*	*Reference*
	Māori	1.81 (1.60–2.04)*	1.13 (0.99–1.29)	1.07 (0.94–1.22)
	Pacific	0.86 (0.68–1.09)	0.45 (0.35–0.58)*	0.46 (0.36–0.59)*
	Asian	0.43 (0.34–0.54)*	0.34 (0.27–0.43)*	0.40 (0.31–0.50)*
	MELAA	0.48 (0.25–0.93)*	0.30 (0.15–0.58)*	0.34 (0.17–0.65)*
	Other	0.18 (0.11–0.27)*	0.38 (0.24–0.61)*	0.41 (0.26–0.65)*
**Marital Status**	Single	*Reference*	*Reference*	*Reference*
	Married	0.44 (0.38–0.51)*	0.44 (0.37–0.52)*	0.43 (0.37–0.51)*
	Civil Union	1.85 (1.06–3.20)*	2.11 (1.22–3.68)*	2.12 (1.22–3.69)*
	Separated	1.43 (1.11–1.84)*	1.29 (0.99–1.67)	1.27 (0.98–1.65)
	Divorced	1.12 (0.92–1.37)	1.08 (0.87–1.34)	1.08 (0.87–1.34)
	Widowed	0.61 (0.44–0.84)*	0.85 (0.59–1.21)	0.87 (0.61–1.24)
	Undefined	2.95 (2.61–3.33)*	7.46 (6.56–8.47)*	8.56 (7.52–9.74)*
**NZIMD Decile (No Health Domain)**	1	*Reference*	*Reference*	*Reference*
	2	1.28 (0.98–1.67)	1.11 (0.84–1.46)	1.17 (0.90–1.52)
	3	1.28 (0.98–1.67)	1.47 (1.13–1.90)*	1.13 (0.87–1.48)
	4	1.45 (1.12–1.88)*	1.34 (1.03–1.74)*	1.24 (0.96–1.61)
	5	1.66 (1.29–2.13)*	1.38 (1.07–1.79)*	1.36 (1.05–1.75)*
	6	1.76 (1.37–2.25)*	1.52 (1.19–1.97)*	1.39 (1.08–1.79)*
	7	1.96 (1.54–2.51)*	1.46 (1.13–1.89)*	1.45 (1.13–1.86)*
	8	1.91 (1.49–2.45)*	1.77 (1.39–2.27)*	1.34 (1.03–1.72)*
	9	2.14 (1.67–2.73)*	1.47 (1.14–1.89)*	1.40 (1.09–1.80)*
	10	2.62 (2.06–3.33)*	1.72 (1.34–2.22)*	1.49 (1.16–1.92)*
	NA	0.09 (0.04–0.20)*	0.02 (0.01–0.04)*	0.04 (0.02–0.09)*
**Contact Period**	No Contact			*Reference*
	0–6 Months			4.43 (3.73–5.24)*
	7–12 Months			1.08 (0.86–1.36)
	13–18 Months			1.41 (1.06–1.87)*
	19–24 Months			1.14 (0.77–1.67)

Co-variates in the base model include sex, age band, ethnicity, marital status, NZIMD (excluding the Health Domain). Full model adjusts for base model variables and contact (with any service type).

These relationships persisted–although attenuated–in the Base Model, which adjusted for age, sex, ethnicity, marital status, and area deprivation (IMD2013). The association between males and suicide was strengthened after controlling for contact with any type of health services in our Full Model (OR 3.41, [95% CI 3.04–3.82]), as compared to the univariate tests (OR 3.02, [95% CI 2.70–3.38]) and Base Model (OR 2.97, [95% CI 2.65–3.322]).

In the univariate analyses, all age groups had a lower odd of suicide compared to the reference group of 15–19 Years. In the Base Model, where we adjust for demographic factors, those aged 30–59 Years have higher odds of suicide compared to individuals aged 15–19 Years. This statistically significant relationship persists after adjusting for any type of health services contact. This can be seen in the Full Model and the three models that adjusted for the three service types of interest (Table 2). Conversely, the odds of suicide for those aged 65–69 years are lower than observed for the 15–19 year age group. This relationship is statistically significant in all but the Base Model. Māori are the only ethnic group to show increased odds of suicide when compared to Europeans in both the univariate and multivariate analyses, although this association was not significant in the base or full models.

Married and widowed individuals have lower odds of suicide, while those in civil union, or are separated or divorced are more like to die of suicide compared to single individuals. Individuals with an Undefined marital status had the highest odds of suicide compared to all other marital status groups. The odds of suicide for each marital status group remain relatively stable with the adjustments seen in Table 2 (or in S1 Table). The greatest change in OR is observed in the Undefined group, where odds of suicide increased from 2.95 [95% CI 2.61–3.33] to 7.46 [95% CI 6.56–8.47] in the Base Model and to 8.56 [95% CI 7.52–9.74] after adjusting for any type of service contact in the Full Model.

There is an increasing risk of suicide as the level of deprivation increases. This relationship is observable in both the univariate and multivariate analyses.

**Fig 3:**
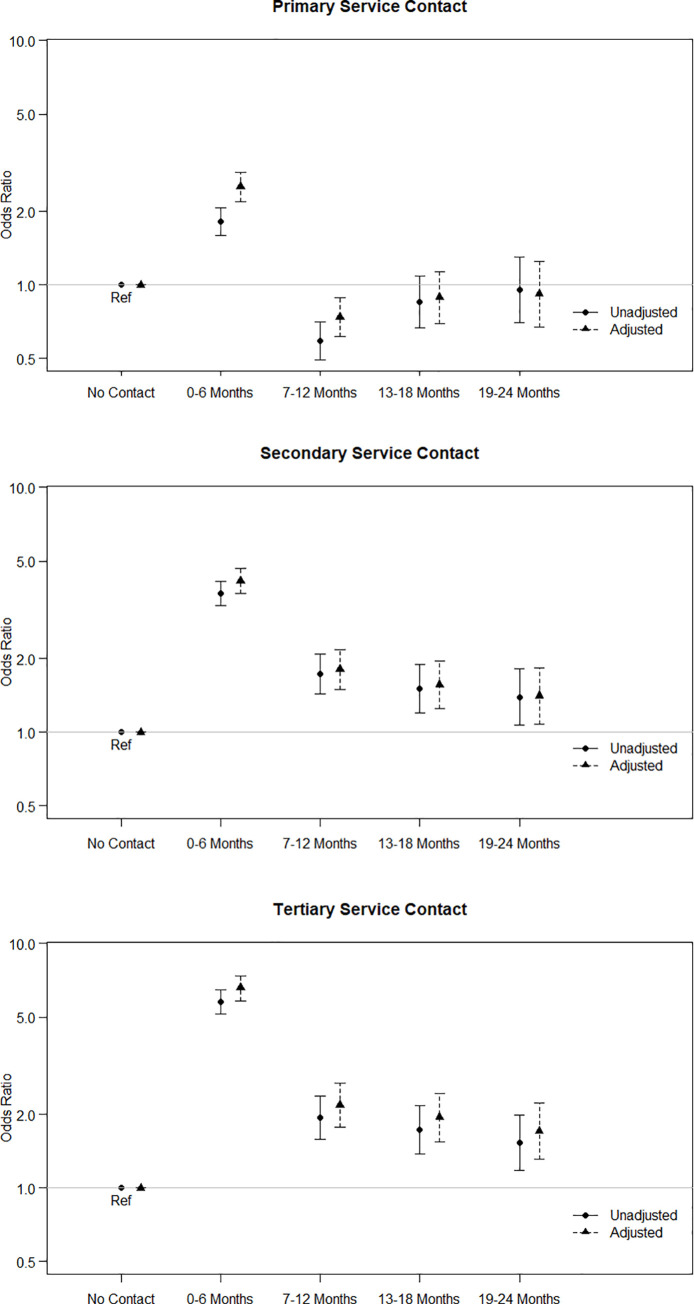
Unadjusted (univariate) and adjusted odds of suicide associated with contact primary (A), secondary (B) and tertiary (C) services. Adjusted odds control for the Base Model variables of sex, age group, ethnic group, marital status and NZIMD (No Health). The adjusted odds ratios are also available in S1 Table.

Contact with each type of health services in the past 6 Months was associated with an increase in the odds of suicide, which might be expected if those at higher risk of suicide are making increased contact with services at a whole. Fig 3 shows the unadjusted odds associated with primary, secondary, and tertiary services, alongside the odds after adjusting for service type and the Base Model. Overall, adjustment for demography and IMD2013, slightly strengthens the associations between suicide and health service contact. Primary services contact was associated with an increased odd of suicide of 2.51 [95 CI% 2.19–2.88] for individuals whose last contact was in the past 6 Months, compared to those that did not have any contact with primary care in the past 24 Months. However, the odds of suicide appear to be 0.74 [95% CI 0.61–0.89] for those that had a primary service contact in the past 7–12 Months, compared to those that did not have any contact. Contact with secondary and tertiary services in the past 6 Months was associated with odds of suicide of 4.45 [95% 3.69–4.66] and 6.57 [95% CI 5.84–7.38], respectively, compared to individuals that did not have contact with these services within 24 Months. The odds of suicide associated with secondary and tertiary are persistently high, these odds decrease as time since last contact with these services increase.

## Discussion

This study suggests that suicide decedents were more likely to have made primary care contact in the past 6 Months and contact with secondary and tertiary services in the past 24 Months than a surviving cohort of people. This association, between health service contact and increased odds of suicide, potentially allows for increased focus on identifying those in crisis. Whilst other studies have considered the relationship of suicide risks to only primary care contact, we found an even stronger relationship exists for contacts with tertiary providers than for primary health care.

Prior to this study, most studies on suicide risks have focussed on sub-populations [11–16] without comparing against the general population. Where whole-of-population approaches have been taken, they have either been interested in the association of suicide and primary contact exclusively [35–37], or in describing the frequency and type of contact that suicide decedents had prior to death [38]. To our knowledge this is the first observational study internationally that uses record linkage to create a whole-of-population assessment of suicide and its association with prior contact compared to the general population.

In both our univariate and multivariate analyses, males had a higher odd of suicide compared to females. Inclusion of service contact time co-variates in analysis strengthened the association between being a suicide decedent and being male, which is consistent with existing evidence from New Zealand that report men under-utilising health services [16]. Our finding is consistent with those of Jatrana and Crampton [39], who found that men were less likely to utilise primary health services, specifically, compared to women. Jatrana and Crampton [39] postulated that the under-utilisation of services by men is perhaps due a lack of awareness or reluctance to acknowledge the severity of the health concerns both prior to and in their encounters with health professionals. Our study is unable to draw causative links between males presenting to health services and suicides, though it contributes to the evidence base that men may seek health care when their health concerns, including mental health, are more severe.

Extensive evidence shows that Māori (and other indigenous populations internationally) are over-represented in suicide and health statistics more generally [7, 40–43]. Our univariate analyses concur with this, however the risk of suicide for Māori is no longer statistically different to the New Zealand European (reference) population once adjusted for service contact and sociodemographic factors. We do not perceive this as an indicator of equity in terms of ethnic disparities in Aotearoa NZ. Rather, these results may suggest low service utilisation among Māori are persistently low despite increased severity of health concerns. Māori have a younger age structure compared to the general population and are disproportionately overrepresented in areas of high deprivation and are nearly twice as likely to cite cost as a barrier to accessing care compared to non-Māori [44]. Māori are also less likely to be referred for specialist care and more likely to report negative experiences when receiving care [43, 45]. There is the possibility that young age and high socioeconomic deprivation, which are themselves associated with high odds of suicide in our regression models, are potentially diminishing the effect size seen in the ethnicity variable.

In all models tested, we found a positive correlation between increasing levels of deprivation and the odds of suicide. This was consistent with the study conducted by John et al [46], which used the similar Welsh Index of Multiple Deprivation.

Linking individuals across routinely collected datasets was a strength of this study and allowed for the imputation of demographic data such as age, sex, and ethnicity, if there was person-level or item-level missingness in either the Census or mortality datasets. The only variable that could not be imputed in this way was marital status. As a result, those who did not complete the Census but whose demographic data could otherwise be identified appear as the ‘Undefined’ marital status category. Whilst social theories on suicide suggest that there are strong links between unstable relationships and suicide, this confounding limits any claim that our study can make with regards to this phenomenon.

The linkage of Census and mortality data in our study produced an unexpected finding, showing that just under one-third of suicide decedents were not counted in the 2013 Census, as compared to only 2.4% of people across New Zealand as a whole according to the Census’ post-enumeration survey. This suggests that suicide decedents are disproportionately undercounted prior to death. Here, Māori were also found to the most likely to be undercounted, with an undercount rate of 6.1%, compared to 1.9% for Europeans. Further research on this relationship could consider parallels between the reasons for non-participation or missingness in civic activities such as the census and how this may be influenced by mental and social wellbeing.

Whilst the odds of suicide associated with primary care are lower compared to the odds associated with secondary and tertiary service, the primary care setting represents the most frequently contacted service type in our study and in similar studies conducted internationally [38, 46]. Reviews of primary care contact found that most suicide decedents are in contact with their primary care provide in the 6–12 months prior to their death, consistent with the findings of this study [12, 37]. We found the greatest odds of suicide was associated with contact with any of the three service types in the past 6 months, which suggests that strategies for the prevention of suicide should not necessarily be confined to primary care but addressed through a systems-wide approach. Nevertheless, the gate-keeper role of primary carers in New Zealand suggests that primary care remains an important setting for coordination and continuity of care. Of concern is the body of literature that relating to a lack of education and support for primary care physicians in how to recognise and manage the care of patients in distress [15, 47, 48]. A recent qualitative evaluation of suicide prevention in primary care within the Netherlands emphasised the need for suicide prevention strategies at primary care to include improved liaison with mental health services and primary care [49]. In a second qualitative study, Leavey et al [50] also suggest that there is concern that GPs in the UK lack confidence in their recognition and management of suicidal patients, with several issues raised around the relationships and information flow between GPs and other parts of the health care system. Concerns were raised, for instance, around GPs feeling that their views were discounted when patients were assessed within secondary/tertiary mental health services.

A limitation of this study was that we did not explore the type of secondary and tertiary care received, or the pattern of contact between the three types of care. Future research in this area could consider the specific service types in secondary and tertiary levels, contact with certain combinations of service types or patterns of repeated contact with health services, to better understand potential co-morbidities for suicidality in a NZ context. Understanding the patterns of service type contact would be a natural progression of our study and could support a similar shift towards and all of service model, as is taking place in Australia [9].

Further research in this area could consider the specific services which suicide patients were being discharged from or the health conditions that lead to the admission. The potential implication of research in this area would be to highlight interactions outside of mental health services where prevention strategies may be effective. Data linkage with other routinely collected datasets, such as justice and welfare, has potential to highlight other contexts that may lead to an increase in suicide risk and point to settings where preventative activities may be beneficial.

## Supporting information

S1 Table Adjusted models for contact with primary, secondary and tertiary care. Ba*s*e Model include sex, age band, ethnicity, marital status, NZIMD (excluding the Health Domain).
(DOCX)

S1 File (XLSX)
