# Supplementary material for: Suicide preceded by health services contact – A whole-of-population study in New Zealand 2013-2015
Source: PLoS One. 2021 Dec 20;16(12):e0261163. doi: 10.1371/journal.pone.0261163 (PMC8687551; doi:10.1371/journal.pone.0261163)
Supplement: S1 Table — Base Model include sex, age band, ethnicity, marital status, NZIMD (excluding the Health Domain). (DOCX) [file pone.0261163.s001.docx]

**Supplementary Table 1: Adjusted models for contact with primary, secondary and tertiary care.** Ba*s*e Model include sex, age band, ethnicity, marital status, NZIMD (excluding the Health Domain)*.*

|  |  | Primary Model  (Base Model +  Primary Contacts) | Secondary  (Base Model +  Secondary Contacts) | Tertiary  (Base Model +  Tertiary Contacts) |
| --- | --- | --- | --- | --- |
| Sex | Female |  |  |  |
|  | Male | 3.28 (2.93-3.68)* | 3.22 (2.87-3.61)* | 3.30 (2.94-3.69)* |
| Age Band | 15-19 |  |  |  |
|  | 20-24 | 1.24 (1.00-1.53) | 1.26 (1.01-1.56)* | 1.21 (0.97-1.50) |
|  | 25-29 | 1.09 (0.86-1.39) | 1.13 (0.89-1.43) | 1.06 (0.84-1.35) |
|  | 30-34 | 1.58 (1.26-1.99)* | 1.64 (1.31-2.06)* | 1.58 (1.26-1.98)* |
|  | 35-39 | 1.45 (1.51-1.84)* | 1.53 (1.21-1.93)* | 1.52 (1.20-1.92)* |
|  | 40-44 | 1.54 (1.23-1.92)* | 1.64 (1.31-2.05)* | 1.66 (1.32-2.07)* |
|  | 45-49 | 1.54 (1.23-1.93)* | 1.63 (1.30-2.03)* | 1.67 (1.34-2.09)* |
|  | 50-54 | 1.34 (1.06-1.69)* | 1.41 (1.11-1.77)* | 1.47 (1.16-1.85)* |
|  | 55-59 | 1.29 (1.01-1.64)* | 1.33 (1.04-1.69)* | 1.39 (1.09-1.78)* |
|  | 60-64 | 0.88 (0.66-1.16)* | 0.89 (0.67-1.18) | 0.94 (0.71-1.24) |
|  | 65-69 | 0.56 (0.39-0.79)* | 0.54 (0.38-0.77)* | 0.56 (0.40-0.79)* |
|  | 70-74 | 0.67 (0.47-0.96)* | 0.63 (0.44-0.89)* | 0.63 (0.44-0.90)* |
|  | 75-79 | 0.72 (0.49-1.07) | 0.65 (0.44-0.97)* | 0.63 (0.42-0.93)* |
|  | 80+ | 1.01 (0.73-1.39) | 0.90 (0.65-1.25) | 0.81 (0.59-1.12) |
| Ethnicity | European |  |  |  |
|  | Māori | 1.11 (0.98-1.27) | 1.04 (0.91-1.18) | 1.05 (0.92-1.20) |
|  | Pacific | 0.46 (0.35-0.58)* | 0.45 (0.35-0.58)* | 0.45 (0.35-0.58)* |
|  | Asian | 0.37 (0.29-0.46)* | 0.38 (0.30-0.48)* | 0.38 (0.30-0.49)* |
|  | MELAA | 0.32 (0.17-0.62)* | 0.31 (0.16-0.60)* | 0.31 (0.16-0.60)* |
|  | Other | 0.40 (0.25-0.63)* | 0.40 (0.25-0.64)* | 0.40 (0.25-0.64)* |
| Marital Status | Single |  |  |  |
|  | Married | 0.44 (0.37-0.51)* | 0.45 (0.98-0.52)* | 0.44 (0.38-0.52)* |
|  | Civil Union | 2.12 (1.22-3.68)* | 2.10 (1.20-3.64)* | 2.07 (1.19-3.60)* |
|  | Separated | 1.27 (0.98-1.66) | 1.25 (0.96-1.63) | 1.23 (0.95-1.60) |
|  | Divorced | 1.08 (0.87-1.33) | 1.07 (0.86-1.32) | 1.05 (0.85-1.30) |
|  | Widowed | 0.85 (0.62-1.21) | 0.85 (0.59-1.21) | 0.85 (0.59-1.21) |
|  | Not Stated | 8.03 (7.05-9.14)* | 7.93 (3.98-9.02)* | 7.74 (6.81-8.80)* |
| NZIMD Decile  (No Health Domain) | 1 |  |  |  |
|  | 2 | 1.17 (0.90-1.52) | 1.14 (0.88-1.49) | 1.15 (0.88-1.50) |
|  | 3 | 1.14 (0.87-1.48) | 1.09 (0.83-1.42) | 1.11 (0.85-1.45) |
|  | 4 | 1.25 (0.96-1.62) | 1.18 (0.91-1.54) | 1.22 (0.94-1.56) |
|  | 5 | 1.37 (1.07-1.77)* | 1.29 (1.00-1.66) | 1.32 (1.03-1.41)* |
|  | 6 | 1.42 (1.10-1.82)* | 1.32 (1.03-1.69)* | 1.36 (1.06-1.44)* |
|  | 7 | 1.49 (1.16-1.91)* | 1.36 (1.06-1.74)* | 1.42 (1.11-1.82)* |
|  | 8 | 1.39 (1.08-1.79)* | 1.25 (0.98-1.61) | 1.31 (1.02-1.70)* |
|  | 9 | 1.45 (1.13-1.86)* | 1.29 (1.01-1.66)* | 1.36 (1.06-1.75)* |
|  | 10 | 1.56 (1.21-2.00)* | 1.38 (1.07-1.77)* | 1.44 (1.12-1.85)* |
|  | NA | 0.02 (0.01-0.06)* | 0.03 (0.01-0.06)* | 0.02 (0.01-0.06)* |
| Contact Period | No Contact | *Reference* |  |  |
|  | 0-6 months | 2.51 (2.19-2.88)* | 4.45 (3.69-4.66)* | 6.57 (5.84-7.38)* |
|  | 7-12 months | 0.74 (0.61-0.89)* | 1.80 (1.49-2.18)* | 2.18 (1.78-2.68)* |
|  | 13-18 months | 0.89 (0.69-1.14) | 1.56 (1.24-1.96)* | 1.94 (1.54-2.44)* |
|  | 19-24 months | 0.92 (0.67-1.25) | 1.41 (1.08-1.84)* | 1.71 (1.31-2.23)* |
